# Controlled fracture of the medial wall versus structural autograft with bulk femoral head to increase cup coverage by host bone for total hip arthroplasty in osteoarthritis secondary to developmental dysplasia of the hip: a retrospective cohort study

**DOI:** 10.1186/s13018-020-02088-5

**Published:** 2020-11-26

**Authors:** Ping Mou, Kai Liao, Hui-lin Chen, Jing Yang

**Affiliations:** 1 Department of Orthopedic Surgery, West China Hospital, West China Medical School, Sichuan University, #37 Guoxue Road, Chengdu, 610041 Sichuan Province People’s Republic of China; 2 Department of Radiology, West China Hospital, West China Medical School, Sichuan University, Chengdu, 610041 Sichuan Province People’s Republic of China; 3 Clinical Medicine, West China Hospital, West China Medical School, Sichuan University, Chengdu, 610041 Sichuan Province People’s Republic of China

**Keywords:** Total hip arthroplasty, Developmental dysplasia of the hip, Cup coverage, Controlled fracture of the medial wall, Structural autograft

## Abstract

**Background:**

Many methods have been proposed to increase cup coverage by host bone during primary total hip arthroplasty (THA) in hip osteoarthritis secondary to developmental dysplasia of the hip (DDH). However, there was no study comparing the results of controlled fracture of the medial wall with a structural autograft with a bulk femoral head.

**Methods:**

Sixty-seven hips classified as Crowe II/III were retrospectively included in this cohort study, which consisted of 33 controlled fractures (group A) and 34 structural autografts (group B). The Harris Hip Scores (HHS) were recorded. The radiological assessments were analyzed. Also, complications are assessed. The paired-sample *t* test was used for data analysis before and after the operation, while the independent sample *T* test was used for the comparison between the two groups. The Pearson chi-square test or the Fisher exact test was used to analyze the qualitative comparative parameters. Kaplan-Meier was utilized in the analysis of survivorship with the end points as a revision for any component.

**Results:**

All patients were reconstructed acetabulum at the anatomical location. HHS increased greatly for both groups (*p* = 0.18). No statistic difference was observed for the two groups in postoperative leg-length discrepancy (0.51 ± 0.29 cm for group A and 0.46 ± 0.39 cm for group B, *p* = 0.64 ), postoperative height of the hip center (2.25 ± 0.42 cm for group A and 2.09 ± 0.31 cm for group B, *p* = 0.13), and inclination of the cup (39 ± 4° for group A and 38 ± 3° for group B, *p* = 0.65 ). The rate of cup coverage for group B (94 ± 2%) was better than for group A (91 ± 5%), (*p* = .009). The rate of cup protrusio was 48 ± 4% for group A. For both groups, no statistical difference was observed in the cup diameter (*p* > .05), while group A showed less operation time than group B (*p* < .001). No complications were observed at the latest follow-up.

**Conclusion:**

Controlled fracture of the medial wall to increase cup coverage by host bone at the anatomical location can act as an alternative technique for DDH Crowe II/III with the advantage of shorter operation time and less technically demanding.

## Introduction

Total hip arthroplasty (THA) in osteoarthritis secondary to developmental dysplasia of the hip (DDH) is challenging [1]. And many studies [2–4] have reported unfavorable clinical outcomes and higher rate of complications of THA in osteoarthritis secondary to DDH compared with primary hip osteoarthritis. Because DDH presents a spectrum of anatomical disorders including the femur and the acetabulum, the abnormal femoral change of the oversize anteversion and narrow medullary cavity can be easily handled by a modular hip stem, which can correct the overanteverted femoral neck and provide rotational stability if subtrochanteric shortening osteotomy was performed [5]. For patients without excessive femoral anteversion and narrow medullary cavity, monobloc stem can act as an alternative technique. And many studies have reported similar results [6–8]. But for the distorted acetabulum especially DDH Crowe II/III, it always manifests with pathomorphologic changes including shallow true acetabulum, formation of a neoacetabulum, and superolateral bony deficiency [1, 9–11], all of which make it more complicated and technically demanding to balance acetabular reconstruction at the anatomical location and rate of cup coverage by host bone [12, 13].

There are three main techniques to increase cup coverage by host bone in primary THA: creation of a high hip center [14, 15], structural autograft with bulk femoral head [16, 17], and medial protrusio technique [13, 18]. Although the creation of a high hip center can simplify the process of acetabulum management and has been widely used for revision acetabular reconstruction, yet many scholars [13, 17, 19] agreed on inserting the acetabular cup into the anatomical hip center due to superior biomechanics, better fixation, and stability. Structural bulk bone grafting is another effective technique suggested by scholars to increase cup coverage by host bone [17, 20]. Bone grafting can restore bone mass and realize a superior rate of cup coverage by superolateral fixation of the processed autologous femoral head. But the complexity of operation techniques and potential risks including resorption or collapse of the graft and aseptic loosening keep it away from us [21]. Medial protrusio technique consisting of medial wall penetration, medial wall osteotomy, and controlled fracture of the medial wall is a series of methods that deepens the acetabulum and insert the acetabular cup with a medial aspect beyond the Kohler’ line to achieve a higher rate of cup coverage. With the technique, bone grafting is not necessary to increase the rate of cup coverage. Zhang et al. [13] and Hartofilakidis et al. [18] have published excellent outcomes and safety of the technique. But currently, there are studies just reporting the clinical efficacy of bulk bone autograft or medial protrusio technique. And no cohort study was published. To our knowledge, this was the first study comparing the results of controlled fracture of the medial wall with structural bone grafting to increase cup coverage by host bone for hip osteoarthritis secondary to DDH Crowe II/III.

We hypothesize that for hip osteoarthritis secondary to DDH Crowe II/III, controlled fracture of the medial wall presents similar results like structural autograft with bulk femoral head to increase cup coverage by host bone at the anatomical location on clinical measurements and radiological evaluations.

## Material and methods

### Study design

The study is a retrospective cohort study performed through the retrieval of information on a hospital information system from January 2007 to December 2014. And the targeted patients were recalled to accomplish the follow-up. Study approval was obtained from the Clinical Trials and Biomedical Ethics Committee of West China Hospital.

### Patient selection criteria

The following are the inclusion criteria: (1) patients diagnosed with end-stage hip osteoarthritis secondary to DDH and scheduled to undergo primary THAs during the target period, (2) classification of DDH belonging to type II or III according to Crowe [2], and (3) patients performed controlled fracture of the medial wall or structural autograft with bulk femoral head to increase cup coverage by host bone by the same senior surgeon (the corresponding author). The following are the exclusion criteria: (1) patients performed THAs due to other reasons, (2) other types of DDH according to Crowe, (3) increasing cup coverage by other methods, (4) THA performed by other senior surgeons, and (5) patients lost to follow-up.

Ultimately, we selected and analyzed the data of the patients performed by controlled fracture of the medial wall (group A) or structural autograft with a bulk femoral head (group B). All analyzed participants were identified as Crowe II/III. And we called up the patients to return to our hospital to complete the follow-up. So, we obtained the latest clinical and radiological data.

### Surgical techniques

All acetabular cups were placed at the true acetabulum. After general anesthesia, all patients were located in a lateral position, the hips were exposed with a posterolateral approach, and then sawing off the femoral heads and resecting the osteophytes and synovium around the joint as well as the soft tissue in the cotyloid notch of the true acetabulum were performed. The acetabular preparation is conducted by standardized reaming (approximately 45° of abduction and 15° of anteversion). And the deepening was continued until the outer surface of the internal pelvic cortex was reached. The acetabulum trial component was then inserted into the acetabulum and placed at the appropriate abduction and anterversion to examine the rate of cup coverage. If the rate was not satisfactory or the initial stability of the cup was not realized, we would perform a controlled fracture of the medial wall or structural autograft with the processed bulk femoral head to increase the rate of cup coverage. The surgical option selected to increase cup coverage was determined by the senior surgeon at the time of surgery based on careful planning on AP radiograph and the degree of anatomical deformity. The senior surgeon made the final decision according to preoperative assessments and clinical experience intraoperatively.

The detailed steps of controlled fracture of the medial wall were described as follows: Firstly, the medial wall was spherically fractured by osteotome with the center in the top of the cotyloid notch and one-third diameter of the anteroposterior dimension of the true acetabulum. Take care not to perforate the internal layer of the periosteum. Secondly, migrate the superior autogenous mud-like cancellous graft to the fractured area and disperse uniformly. Thirdly, the cementless acetabular component was placed with appropriate orientation and pressure. At last, before placing the liner, examine the initial stability of the component. If the stability was not satisfactory, supplemental screws would be used to reinforce the early cup stability.

The detailed steps of structural autograft with bulk femoral head were described like Kim and Kadowaki [17]. In brief, firstly, the cancellous surface of the resected bulk femoral head was prepared, and the femoral head was shaped to accommodate bone deficiency. Secondly, the pseudoacetabular floor was reamed to expose the cancellous bone. Thirdly, both cancellous surfaces were impacted with screws to prevent micromotion between the graft and the host bone. At last, insert the cementless acetabular component and check the stability of the component.

For the management of the femur, after expanding the medullary cavity routinely, place the trial component and femoral head. Then, try to realize hip joint reduction and check out the stability on different directions and assess sciatic nerve tension by palpation. For the condition that it was difficult to realize hip joint reduction or excessive tension of the sciatic nerve, we would perform transverse subtrochanteric shortening osteotomy. Then, after finishing the procedures, we would evaluate the range of motion (ROM) of the hip, limb length, and nerve tension. Once all of these were satisfactory, we finally placed the components. Ultimately, irrigated the articular cavity, placed a drainage if transverse subtrochanteric shortening osteotomy was performed, and sutured the incision.

### Perioperative regimen

For all patients, positive motion exercises were initiated on the bed after recovering from anesthesia. Prophylactic intravenous antibiotics were used within the first 24 h postoperatively. Additionally, low-molecular-weight heparin (LMWH) and painkillers were systematically managed to prevent deep venous thrombosis (DVT) and relieve pain, respectively. The drainage tube was removed within 24 h.

From the first postoperative day on, all of the patients were allowed to partial weight-bearing exercises with the help of walker aid and full weight-bearing exercises after 6 weeks. Once the patients can realize independent walking, the walker aid can be removed. For the ones receiving transverse subtrochanteric shortening osteotomy, internal and external rotations of the hip were forbidden until the bony union of the osteotomy.

### Clinical measurements

Clinical details were recorded including operation time, cup diameter, ROM of the hip, and Harris Hip Scores (HHS) [22]. The operation time was defined as the time from skin incision to skin suturing. ROM consisting of flexion, extension, and abduction and Harris scores were examined by 2 authors. Postoperative HHS (the total score is 100) are defined as excellent (> 90), good (80–89), fair (70–79), and poor (< 70).

### Radiological assessments

**Fig. 1:**
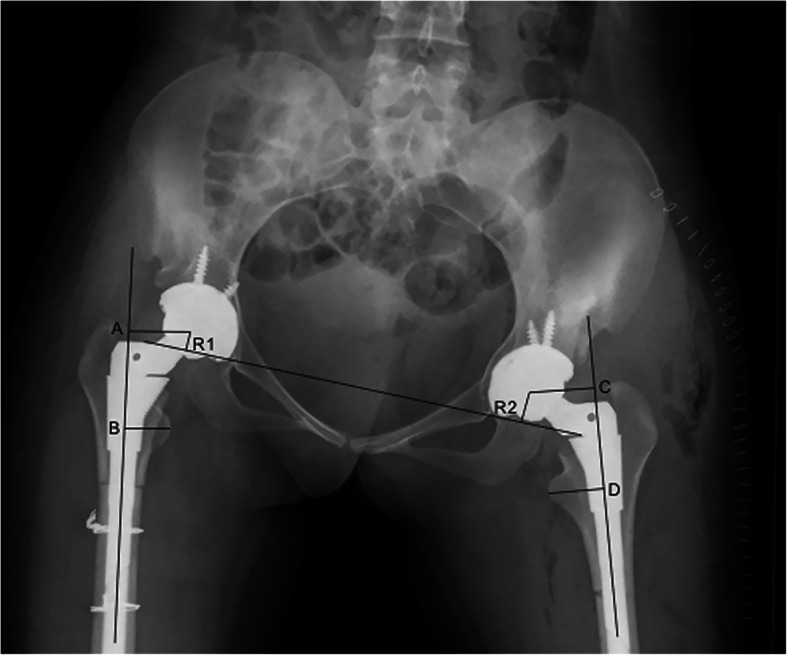
Diagram showing a standardized-trochanteric method to assess leg-length discrepancy. R1 and R2 are the vertical distance from the bilateral center of rotation to the inter-teardrop line. Line AB and line CD are the anatomical axes of the femurs. Point A and point C are the perpendicular intersections from the center of rotation to the femoral anatomical axis. Point B and point D are the perpendicular intersections from the tip of the lesser trochanter to the femoral anatomical axis. H1 and H2 are equal to AB and CD, respectively. Leg-length discrepancy = (H1 − R1) − (H2 − R2)

**Fig. 2:**
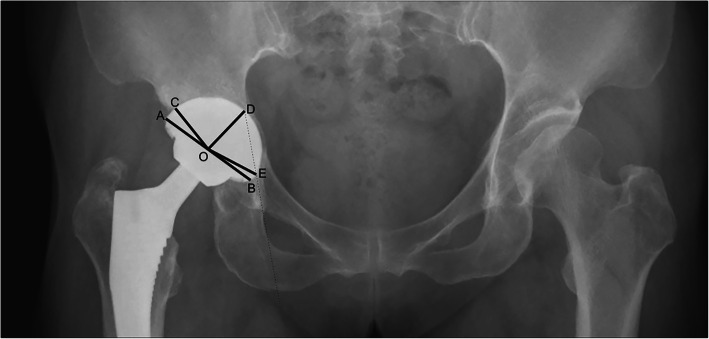
Schematic showing the measurement of medial protrusion and cup coverage. Point O is the center of the cup component. AB is the diameter of the cup component. Point C is the intersection between the edge of the cup implant and the ilium. Point D and point E are the intersections between the medial edge of the acetabular component and Kohler’s line. The rate of cup coverage = [(180° − ∠AOC)/180°] × 100%. The rate of medial protrusion = (∠DOE/180°) × 100%

Standard AP radiograph was obtained before and after surgery. Preoperatively and postoperatively, radiographs were analyzed by 2 authors. The assessments included leg-length discrepancy (LLD), the height of the hip center, inclination of the cup, rate of the cup coverage, and rate of the medial protrusion. LLD was assessed by the standardized-trochanteric method to avoid the influence of pelvic and femoral inclination on the radiographs [23]. The standardized-trochanteric method requires the vertical distance from the inter-teardrop line to the center of rotation and the femoral vertical distance (center of rotation to the lesser trochanter) reference to the femoral anatomical axis. So, the unilateral distance is defined as the difference of the two vertical distances. And LLD is equal to the difference of the two unilateral distances (Fig. 1). The height of the hip center is defined as the perpendicular distance from the femoral head center to the inter-teardrop line [24]. The inclination of the cup is defined as the angle between a horizontal line joining the ischial spines and a line parallel to the opening plane of the cup [25]. The rate of cup coverage and the rate of medial protrusion are measured according to the methods introduced by Dorr et al. [26] and Kim et al. [27]. The rate of the cup coverage is defined as the ratio of the degree of the cup covered by the host bone and 180° (Fig. 2). The rate of the medial protrusion is defined as the ratio of the degree of cup medialization beyond the Kohler’s line and 180° (Fig. 2).

### Complications

The complications are recorded including early-onset and late-onset complications during the period of perioperation and follow-up. The early-onset ones consist of infection, intraoperative fracture, DVT, pulmonary embolism, and nerve palsy. Meanwhile, the late-onset ones consist of postoperative dislocation, nonunion of the femoral osteotomy, graft collapse, polyethylene wear, osteolysis, and aseptic loosening [28–30].

### Statistical analysis

Statistical analysis was performed using the SPSS software for Windows version 22.0 (SPSS, Chicago, IL). The level of statistical significance was set at *p* < 0.05. The results were expressed as the mean ± standard deviation. The paired-sample *t* test was used for data analysis before and after the operation, while the independent sample *T* test was used for the comparison between the two groups. The Pearson chi-square test or the Fisher exact test was used to analyze the qualitative comparative parameters. Kaplan-Meier was utilized in the analysis of survivorship with the end points as a revision for any component.

## Results

For group A, the mean age of all patients (male:female = 4:27) was 49.2 years (49–67), and the mean body mass index (BMI) was 22.8 kg/m^2^ (17.2–27.4). Besides, the patients (33 hips) were analyzed with 12 hips classified as type II and 21 hips classified as type III according to Crowe. For group B, the mean age of all patients (male:female = 6:24) was 50.9 years (33–63), and the mean BMI was 22.9 kg/m^2^ (17.3–29.7). Moreover, the patients (34 hips) were analyzed with 10 hips classified as type II and 24 hips classified as type III according to Crowe. These patients underwent 67 THAs performed by the same senior surgeon. Five patients of group A and 6 patients of group B were performed transverse subtrochanteric shortening osteotomy. All patients were followed up using a standard clinical and radiographic protocol mentioned above. Also, we recorded the information about the components. And the related details were presented in Table 1. All implants of both groups used during the procedures were from DePuy, Warsaw, IN. The patients requiring THAs on both hips were performed separately.

**Table 1:** Baseline of all recruited patients

DDH (no. = 67 hips)	Group A (no. = 33 hips)	Group B (no. = 34 hips)
Mean age (years)	49.2 ± 8.3 (range, 49–67)	50.9 ± 9.1 (range, 63–33)
Male:female	4:27	6:24
Mean height (cm)	157.4 ± 6.2 (range, 149–175)	155.0 ± 6.5 (range, 145–170)
Mean weight (kg)	56.3 ± 6.7 (range, 43–70)	54.8 ± 7.6 (range, 43–68)
Mean BMI (kg/m^2^)	22.8 ± 2.6 (range, 17.2–27.4)	22.9 ± 3.3 (range, 17.3–29.7)
Crowe classification		
II	12	10
III	21	24
Follow-up (months)	85 ± 36	78 ± 35
Cup type		
Pinnacle	33	34
Stem type		
Corail	15	10
Tri-lock	3	3
S-rom	15	21
Friction couples		
Ceramic-on-ceramic	28	30
Ceramic-on-polyethylene	5	4
Subtrochanteric osteotomy		
Yes/no	5/28	6/28

*DDH* Developmental dysplasia of the hip

### Clinical outcomes

Ultimately, 61 patients (33 hips of group A and 34 hips of group B) were followed up for 85 ± 36 months for group A and 78 ± 35 months for group B. All patients were satisfied with the results that the pain had decreased and gait had improved markedly compared with preoperative status. For group A, the average HHS improved from 38 ± 6 points preoperatively to 87 ± 6 points at the latest follow-up. According to the postoperative HHS, 15 hips (45.5%) are defined as excellent, 13 hips (39.4%) good, and 5 hips (15.2%) fair. The average flexion, extension, and abduction of the hip increased from 88 ± 25°, − 1 ± 3°, and 20 ± 11° preoperatively to 113 ± 7°, 0 ± 0°, and 38 ± 4° at the final follow-up, respectively. For group B, comparably, the average HHS improved from 40 ± 4 preoperatively to 89 ± 6 at the latest follow-up. According to the postoperative HHS, 17 hips (50%) are defined as excellent, 12 hips (35.3%) good, and 5 hips (14.7%) fair. The average flexion, extension, and abduction of the hip increased from 91 ± 16°, − 2 ± 3°, and 23 ± 6° preoperatively to 116 ± 5°, 0 ± 0°, and 36 ± 3° at the latest follow-up, respectively (Table 2). The mean operation time of both groups showed a statistical difference (*T* = − 6.49, *p* < .001). Group A was 76.8 (51–125) min, while group B was 107.2 (80–143) min (Table 3). The outer and inner diameters of the cup in group A were 46 (44–50) and 29.7 (28–36) mm, while that of group B were 46.5 (44–52) and 29.5 (28–36) mm, respectively, both of which showed no statistical difference (*p* > .05) (Table 3).

**Table 2:** Range of motion and Harris Hip Scores for all recruited patients preoperatively and postoperatively of both two groups

DDH	Group A		Group B		Intra		Inter	
	Pre	Post	Pre	Post	Group A	Group B	Pre	Post
Flexion (°)	88 ± 25	113 ± 7	91 ± 16	116 ± 5	*t* = − 5.86, *p* < .001*	*t* = − 9.97, *p* < .001*	*T* = − 0.55, *p* = .59	*T* = − 1.37, *p* = .18
Extension (°)	− 1 ± 3	0 ± 0	− 2 ± 3	0 ± 0	*t* = − 1.75, *p* = .095	*t* = − 2.30, *p* = .031*	*T* = 0.26, *p* = .54	–
Abduction (°)	20 ± 11	38 ± 4	23 ± 6	36 ± 3	*t* = − 10.29, *p* < .001*	*t* = − 12.88, *p* < .001*	*T* = − 1.08, *p* = .29	*T* = 1.58, *p* = .12
HHS	38 ± 6	87 ± 6	40 ± 4	89 ± 6	*t* = − 65.35, *p* < .001*	*t* = − 77.36, *p* < .001*	*T* = − 1.24, *p* = .22	*T* = − 1.36, *p* = .18

*Pre* Preoperatively, *Post* Postoperatively, *HHS* Harris Hip Scores, *Intra* Intra-group comparisons, *Inter* Inter-group comparisons, *DDH* Developmental dysplasia of the hip

*p values with statistical significance

**Table 3:** Comparison of radiographic outcomes postoperatively and clinical outcomes of all included patients

DDH	Group A	Group B	Inter
IC (°)	39 ± 4°	38 ± 3°	*T* = 0.46, *p* = .65
RCC (%)	91 ± 5%	94 ± 2%	*T* = − 2.78, *p* = .009*
Outer diameter of the cup (mm)	46.8 ± 2.0	46.5 ± 1.8	*p* = .111
Inner diameter of the cup (mm)	29.7 ± 2.2	29.5 ± 2.2	*p* = .94
Operation time (min)	76.8 ± 20.9	107.2 ± 17.2	*T* = − 6.49, *p* < .001*

*IC* Inclination of cup, *RCC* Rate of cup coverage, *Inter* Inter-group comparisons, *DDH* Developmental dysplasia of the hip

*p values with statistical significance

### Radiological outcomes

**Fig. 3:**
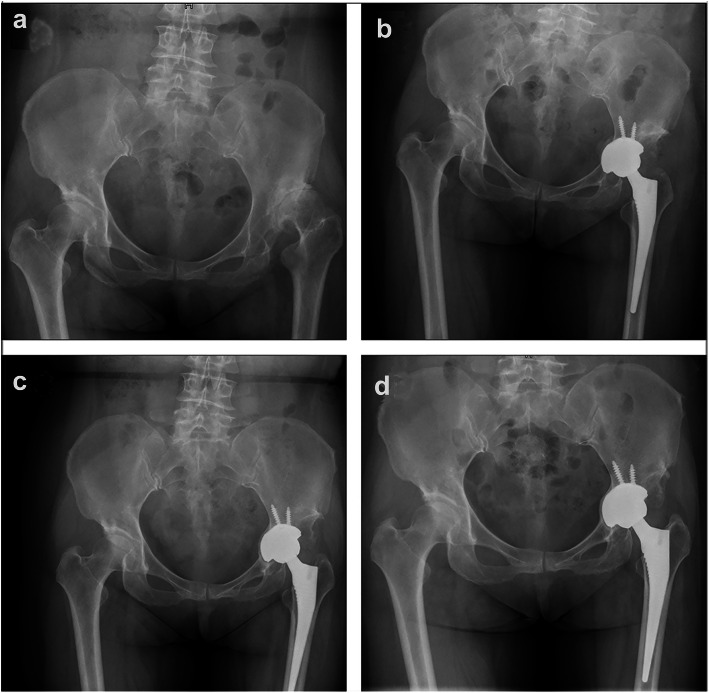
Case presentation of controlled fracture of the medial wall for THA. **a** A 54-year-old woman was diagnosed with DDH Crowe III on the radiograph of the pelvis preoperatively. **b** The radiograph of the pelvis after surgery immediately showed the rate of cup coverage was 86%, the rate of medial protrusion was 52%, and the leg-length discrepancy was 0.38 cm. **c** The radiograph of the pelvis at 1-year follow-up showed the medial wall was a bony union and no aseptic loosening of the component. **d** The radiograph of the pelvis at 76-month follow-up showed no aseptic loosening and migration of the component

**Fig. 4:**
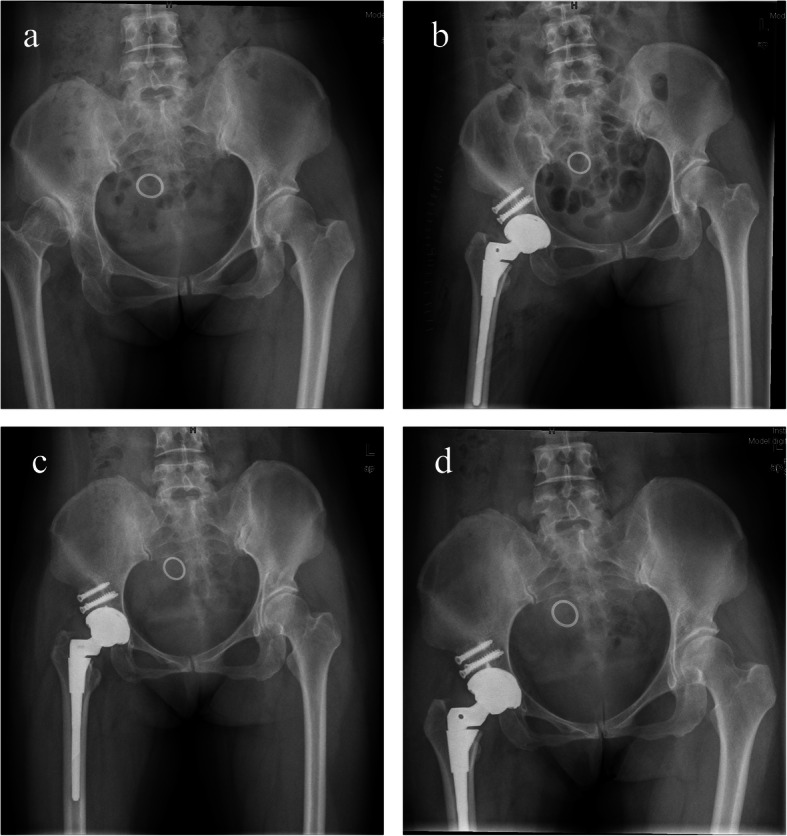
Case presentation of structural autograft with a bulk femoral head for THA. **a** A 51-year-old woman was diagnosed with DDH Crowe III on the radiograph of the pelvis preoperatively. **b** The radiograph of the pelvis after surgery immediately showed the rate of cup coverage was 95% and the leg-length discrepancy was 0.16 cm. **c** The radiograph of the pelvis at 1-year follow-up showed the interface between the graft and the host bone had been obscure and no aseptic loosening of the component. **d** The radiograph of the pelvis at 84-month follow-up showed incorporation of grafted bone and no aseptic loosening and migration of the component

For group A, at the latest follow-up, LLD restored from 2.31 ± 1.65 cm preoperatively to 0.51 ± 0.29 cm. The height of the hip center was restored from 4.34 ± 1.03 to 2.25 ± 0.42 cm. The inclination of the cup was 39 ± 4°. The rate of the cup coverage was 91 ± 5%. And the rate of the cup protrusio was 48 ± 4% (Tables 3 and 4) (Fig. 3). Additionally, for group B, at the final follow-up, LLD restored from 2.46 ± 1.37 to 0.46 ± 0.39 cm. The height of the hip center restored from 4.59 ± 0.59 to 2.09 ± 0.31 cm. the inclination of the cup was 38 ± 3°. The rate of the cup coverage was 94 ± 2% (Tables 3 and 4) (Fig. 4). For the patients performed transverse subtrochanteric shortening osteotomy of both two groups, most of them (10 of 11) realized a bony union at 6 months after surgery, and the remaining one realized at 9 months postoperatively. From the X-ray films of the latest follow-up, we did not find radiolucent lines, visible implant loosening, and periprosthetic osteolysis for both groups. Moreover, we observed no patient encountering graft collapse or progressive migration of the implant from the radiographs.

**Table 4:** Comparison of leg-length discrepancy and height of the hip center by radiography preoperatively and postoperatively of all included patients

DDH	Group A		Group B		Intra		Inter	
	Pre	Post	Pre	Post	Group A	Group B	Pre	Post
LLD (cm)	2.31 ± 1.65	0.51 ± 0.29	2.46 ± 1.37	0.46 ± 0.39	*t* = 4.69, *p* < .001*	*t* = 6.52, *p* < .001*	*T* = − 0.32, *p* = .75	*T* = 0.48, *p* = .64
HHC (cm)	4.34 ± 1.03	2.25 ± 0.42	4.59 ± 0.59	2.09 ± 0.31	*t* = 8.66, *p* < .001*	*t* = 22.56, *p* < .001*	*T* = − 1.02, *p* = .31	*T* = 1.54, *p* = .13

*Pre* Preoperatively, *Post* postoperatively, *Intra* Intra-group comparisons, *Inter* Inter-group comparisons, *LLD* Leg-length discrepancy, *HHC* Height of the hip center, *DDH* Developmental dysplasia of the hip

*p values with statistical significance

### Complications

None of the patients suffered from early-onset complications. But 2 hips of group A and 2 hips of group B (3 hips classified as Crowe III and 1 hip classified as Crowe II) encountered intraoperative fracture of the proximal femur, which were addressed well by wires. For the late-onset complications such as dislocation, osteolysis and radiolucent lines were not observed regardless of the surgical methods and Crowe classification. However, 1 hip classified as Crowe III from group B performed transverse subtrochanteric shortening osteotomy suffered from delay union and finally achieved bony union at 9 months postoperatively. The latest radiograph of the patient showed excellent stability of the acetabular cup and stem. No revision of all patients was required during the follow-up period, although there were apparent differences in bone stock and upper migration between Crowe II and III. And the survival rate of the prosthesis was 100% regardless of the surgical methods and Crowe classification.

## Discussion

The most important finding of this study was that the clinical measurements and radiological assessments of both two groups almost restore to normal, and no one showed complications at the latest follow-up. Besides, for the patients, the pain has relieved and the gait has improved as well as no complaining about self-perceived LLD. So, both two approaches are effective to increase cup coverage by host bone and reconstruct acetabulum. According to the current literature [10], because of abnormal anatomy, secondary osteoarthritis of DDH occurs at a relatively young age (an average of 42 years old), and for the younger population, the long-term survival of implant was reported to be lower than that of the general ones due to more activity [31, 32]. Besides, for DDH Crowe II/III, the biggest challenge lies in cup coverage and acetabular reconstruction. Also, the study [33] has demonstrated that the rate of failure of the acetabular component showed a positive correlation with the severity of DDH. So, the surgeons are more concerned about how to obtain adequate initial stability of the cup in order to realize long-term survival of the acetabular component and postpone potential revision surgery.

There is no consensus on the position of the acetabular component. And the placement of the acetabular implant can be located in the true acetabulum or high hip center. The high hip center is defined as the perpendicular distance from the femoral head center to the inter-teardrop line more than 35 mm [24]. Some scholars [11, 13, 17, 19, 34] agreed on inserting the cup into the true acetabulum due to superior biomechanics, better fixation, and more bone mass. Placing the implant at the anatomical center of the hip enables optimal abductor muscle function, and the bone mass for fixation of the component is larger than at a more proximal level [12]. However, we must address problems such as limb lengthening [12], nerve palsy [12], and less coverage of the cup [19]. Gratifyingly, subtrochanteric osteotomy [10], structural autograft [17], and medial protrusio technique [11] have provided novel and valid methods. Additionally, the creation of a high hip center was also proposed for primary acetabular reconstruction and was reported to have good long-term outcomes and showed no difference in polyethylene wear [15, 35]. Nevertheless, there are many other problems to face. Firstly, high acetabular reconstruction often results in high, lateral, and oversized cup placement leading to the problems like fixation, primary stability, and restoration of normal hip biomechanics [10]. Secondly, at this high level, the bone stock is insufficient, and shearing forces on the acetabular component may lead to early loosening. Meanwhile, a longer lever arm for body weight can result in excessive load to the hip joint [19, 36]. Thirdly, longer prosthetic neck length used to balance leg lengths possibly leads to neck-liner impingement [13]. Lastly, the patients performed primary THA owing to DDH usually are younger than hip osteoarthritis, and most of them likely need revision surgery, which may be more difficult owing to limited bone stock [17]. Bicanic et al. [37] reported that every millimeter of lateral displacement of the acetabular cup compared with the ideal rotation center resulted in an increase of 0.7% in hip load and every millimeter of proximal displacement an increase of 0.1% in hip load. This accounts for a high rate of failure if the cup component is placed in a high hip center. Additionally, Chen et al. [14] and Stans et al. [38] have demonstrated that using a high hip center during acetabular reconstruction in DDH patients had a higher failure rate. So, based on these reasons, we have chosen anatomic placement of the acetabular cup. And the results of this study were that LLD was approximately 5 mm and no one complained about self-perceived LLD, if we reconstructed the acetabulum at the true location. Also, aseptic loosening was not found at the latest follow-up. These results demonstrated the anatomic reconstruction of the acetabulum was a superior choice once again.

**Fig. 5:**
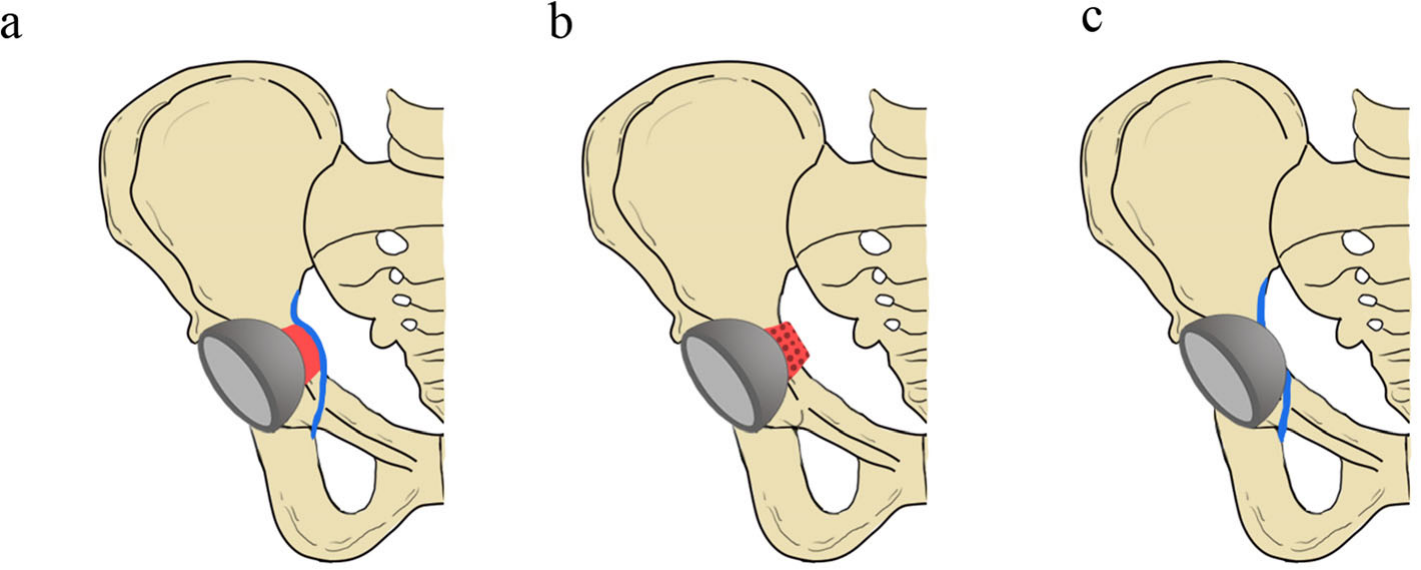
Diagram showing the differences between the three medial protrusion techniques increasing the rate of cup coverage. **a** The illustration of a medial wall fracture. The blue color showed the complete internal layer of the periosteum. The red color showed the autogeneous mud-like cancellous graft and fractured medial wall. **b** The illustration of medial wall osteotomy. The red color showed the medially displaced bone block. **c** The illustration of medial wall penetration. The medial aspect of the acetabular cup beyond the Kohler’s line with a discontinuous internal layer of the periosteum colored in blue and bone loss

Currently, the literatures [18, 39] have proved the medial protrusio technique was an effective method to increase the rate of the cup coverage without a bulk femoral head autograft. The medial protrusio technique includes controlled medial wall fracture, medial wall osteotomy, and medial wall penetration [11]. And the reasons why we chose controlled medial wall fracture as a cotyloplasty are the simplification and safety compared with the other two techniques. Besides, the current literatures [18, 39] have demonstrated that the long-term survival of THA performing controlled fracture of the medial wall showed no difference compared with that of general THA at 10 years. For medial wall osteotomy, it is a more technically demanding process, and the thickness of the medial wall should be not less than 10 mm according to Zhang et al. [13]. For medial wall penetration, the loss of bone stock is more than the medial wall fracture. And it is easy to ream excessively leading to the damage of the acetabular rim. We have provided an illustration (Fig. 5) of the comparison of three medial protrusion techniques. And we hope better explanations can be realized for the differences. To our knowledge, there is no report for more than 10 years about the medial wall osteotomy and medial wall penetration demonstrating comparable outcomes with controlled medial wall fracture. So, based on these factors, we thought controlled medial wall fracture is a better one for increasing cup coverage in THA with DDH.

Sufficient initial stability of cementless cup is imperative for successful osseointegration and good long-term survival, but medial protrusion may decrease this stability. So, what rate of medial protrusion of the cup is the best? Dorr et al. [26] recommended this rate should be less than 45%, and Kim et al. [27] suggested it should be within 50–60%. Besides, according to Zha et al. [11], a prospective 6– to 9-year follow-up of 43 consecutive patients using the medial protrusio technique in cementless THA for DDH demonstrated that the rate of medial protrusion more than 60% had a high aseptic loosening rate in the midterm. Also, the excessive rate of medial protrusion may possibly result in cup component migration into the pelvis. And the rate of medial protrusion of our study was 48 ± 4%, and no complications were observed at the final follow-up. The purpose of medial protrusion was to increase cup coverage by host bone and realize initial cup stability. So, we considered it was reasonable to realize enough cup coverage with the least rate of the medial protrusion. According to the published studies and the results of our research, we suggested that the rate of medial protrusion should be less than 60%. If needed, supplementary screws can be used for initial cup stability. Additionally, the amount of protrusion was evaluated by preoperative templating on an AP radiograph. Also, the surgeon would conduct assessment again intraoperatively after standard acetabular reaming. Preoperative planning would provide a reference for the intraoperative amount of medial protrusion. And the operators can judge the amount of medial protrusion according to intraoperative fluoroscopy compared with preoperative planning. The final amount of medial protrusion was determined on the preoperative planning and intraoperative evaluation.

For patients diagnosed with DDH, the abnormal femoral morphologies contribute to the complexity of the cases, which include excessive femoral anteversion, coxa valga, and small diaphyseal diameters [10]. For these cases, modular stem-like SROM stem (Depuy, Warsaw, IN) may be needed. Compared with monobloc stem, modular stem provides intraoperative flexibility in femoral reconstruction. Sleeves with various height and width options can be chosen to accommodate different metaphyseal morphologies. Also, the different combinations of the neck and stem can optimize offsets, avoid LLD, and match different medullary cavity [40, 41]. If subtrochanteric shortening osteotomy was performed, modular stem-like SROM stem can provide rotational stability [5]. In brief, modular stem possesses advantages including accommodating abnormal femoral medullary cavity, optimizing offset, avoiding LLD, and providing rotational stability. However, there are concerns associated with modular stem including junction failure and corrosion due to the slippage and micromotion that occurred at the stem-sleeve interface [38, 42, 43]. The study from Bobyn et al. demonstrated that the wear particle was not significant enough to cause osteolysis and loosening [44]. Also, Seufert and McGrory [43] and Kong et al. [45] have reported modular stem can successfully accommodate the distorted anatomy of the proximal femur and achieved optimal stem version as well as excellent clinical outcomes. So, modular stem can be recommended as an alternative choice to reconstruct the femora in THA for patients diagnosed with DDH.

The technique, controlled fracture of medial wall, has several advantages to deal with unsatisfying cup coverage compared with structural autograft with a bulk femoral head. Firstly, it can simplify the operation and does not ask for special surgical instruments. Meanwhile, this technique does not prolong the operation time and cause obviously additional damage to the patients. Secondly, the area of operated medial wall belongs to the bony union because of the application of autogenous mud-like cancellous graft and protection of the internal layer of the periosteum. And the integrity of the acetabular rim does not damaged. So, there is little influence on the primary stability and bone ingrowth of the cup component. Thirdly, medialization of the cup component can increase the rate of the cup coverage and decrease wear due to the increase of the abductor lever arm and decreased loading of the hip joint. However, the primary concern of this technique is possibly excessive medial protrusio resulting in unsatisfied primary stability of the cup component or disastrous migration of the cup into pelvis when postoperative weight-bearing exercises. Besides, the range of medial fracture is not easy to control. And The future revision arthroplasty in the patients operated with controlled fracture of the medial wall is another concern, especially the higher fixation problem and bone deficiency. For the mentioned concerns, we suggest that firstly do not chase for an excessive rate of medial protrusio if the cup component can realize enough rate of cup coverage and initial stability. Secondly, the application of autogenous mud-like cancellous graft and protection of the internal layer of the periosteum play a vital role in the bony union of the medial wall. Postoperative function exercise should be rational and not too be ambitious. Thirdly, the man-made fracture of the medial wall could realize bony union by application of autogenous mud-like cancellous graft and protection of the internal layer of the periosteum. So, no excessive bone loss was compared with primary THA. Additionally, with the development of material science, we believe many other effective methods can handle the fixation problem and bone deficiency easily.

The limitations of the current study were the retrospective research with small population and relatively short period of follow-up, and we could not acknowledge the long-term outcomes. Additionally, when we conducted radiological assessments, we just used 2-dimensional images to accomplish evaluations, which might compromise the robustness of the final results. But, it was the first study to compare the effectiveness of controlled fracture of the medial wall with that of structural bone grafting to increase cup coverage and reconstruct acetabulum. And the study demonstrated that a controlled fracture of the medial wall could increase the rate of cup coverage without technically demanding, which can act as a selectable method to increase cup coverage and reconstruct acetabulum.

## Conclusion

Controlled fracture of the medial wall is an effective and safe technique to increase the rate of cup coverage for THA in osteoarthritis secondary to DDH Crowe II/III. With the advantage of less technical demand, we recommended it as an alternative technique to increase cup coverage by host bone for THA in hip osteoarthritis secondary to DDH Crowe II/III.

## Data Availability

The datasets used and/or analyzed during the current study are available from the corresponding author on reasonable request.

## Abbreviations

THA: Total hip arthroplasty
DDH: Developmental dysplasia of the hip
ROM: Range of motion
LMWH: Low-molecular-weight heparin
DVT: Deep venous thrombosis
HHS: Harris hip scores
LLD: Leg-length discrepancy
